# Host Developmental Stage and Vegetation Type Govern Root EcM Fungal Assembly in Temperate Forests

**DOI:** 10.3390/jof11040307

**Published:** 2025-04-11

**Authors:** Dong-Xue Zhao, Yu-Lian Wei, Zi-Qi You, Zhen Bai, Hai-Sheng Yuan

**Affiliations:** 1 CAS Key Laboratory of Forest Ecology and Silviculture, Institute of Applied Ecology, Chinese Academy of Sciences, Shenyang 110016, China; 2 University of the Chinese Academy of Sciences, Beijing 100049, China

**Keywords:** ectomycorrhizal (EcM) fungi, broadleaf, coniferous, juvenile, adult, neutral theory modeling

## Abstract

Ectomycorrhizal (EcM) fungi are critical mediators of forest succession, yet the relative contributions of stochastic (neutral) and deterministic (niche-based) processes in shaping their communities are still poorly understood. We investigated the assembly processes in root EcM fungal communities across juvenile and adult coniferous (*Abies nephrolepis*, *Picea jezoensis*, and *Pinus koraiensis*) and broadleaf (*Acer mono*, *Betula platyphylla*, and *Quercus mongolica*) tree species in northeastern China. Employing neutral theory modeling, alpha and beta diversity metrics, and a random forest analysis, we identified patterns of EcM fungal community assembly and the specific taxa associated with developmental stages of various hosts. Neutral processes contributed to the variation in fungal communities, with adult trees showing a higher explanation power (more than 33% of variation) compared to juvenile trees (less than 7% of variation), reflecting a successional shift in assembly mechanisms. Dispersal dynamics was pronounced in juveniles but diminished with host age. Additionally, alpha diversity increased with host age and was slightly moderated by host identity, while beta diversity reflected stronger effects of host age (PERMANOVA R^2^ = 0.057) than host identity (R^2^ = 0.033). Host age and identity further structured communities, with distinct taxa varying between juvenile vs. adult, and coniferous vs. broadleaf hosts. Our results demonstrate that host maturity drives a transition from deterministic to stochastic assembly, modulated by tree species identity, improving our understanding of plant–fungal dynamics during forest succession.

## 1. Introduction

Ectomycorrhizal (EcM) fungi form symbiotic structures with roots, assisting host plants acquiring nutrients (e.g., nitrogen) in exchange of photosynthesized carbon [1]. EcM fungi are essential tree symbionts, playing a crucial role in nutrient cycling, plant–soil feedback, and overall forest ecosystem stability [2,3,4]. Despite their ecological significance, the mechanisms governing EcM fungal community assembly remain debated, especially in the context of forest succession [5,6,7,8]. While neutral theory posits that stochastic processes—such as dispersal limitation and ecological drift—predominantly shape community structure, niche-based theory emphasizes deterministic factors such as environmental filtering and host specificity [9,10,11,12]. Host plant development modulates the balance between neutral and niche-based processes in fungal community assembly. For example, host maturity favors successional recruitment of new taxa and increased dispersal of diverse taxa with distinct traits, displaying a shift in EcM fungal community assembly from deterministic to neutral processes [13,14]. Understanding the mechanisms influencing these processes is critical to elucidating the development of EcM fungal communities across different host trees and their role in forest succession [6,15,16].

Host age and identity are key determinants of EcM fungal community composition and diversity, as they are closely tied to dynamic plant–soil feedback during forest succession [17,18,19,20,21]. Juvenile trees, with their limited root systems and specialized microhabitats, may favor niche-based process in shaping fungal communities [22,23]. In contrast, adult trees, benefiting from well-developed mycorrhizal networks, prolonged biotic–abiotic interactions, and host–fungal coevolution, may experience a shift toward neutral processes [24,25,26]. The family Pyronemataceae form ectomycorrhizas with late-successional host trees, hydrolyzing complex substances and resisting root pathogens [27]. In addition, *Hebeloma* and *Tomentella* abundances decrease from premature to adult host stages (10 to 100 years old) [28]. *Tomentella* is more abundant in late-stage vegetation (e.g., *Ulmus laciniata*), while *Russula* is more abundant in early-stage vegetation (e.g., *Betula platyphylla*) [29]. Furthermore, the distinct litter chemistry and root exudates of different tree species impose specific selective pressures on EcM fungi [30]. Coniferous litter, which is highly recalcitrant, can significantly alter soil chemistry by increasing alkyl/O-alkyl or aromatic/O-alkyl C ratio and thus accumulating stable and humiliated organic substances. This has pronounced impact on soil microbial activity and community structure compared to broadleaf litter, which is more easily decomposed [31,32]. These differences potentially lead to divergent successional trajectories of EcM community composition across various tree species (e.g., coniferous vs. broadleaf) [28]. For instance, EcM fungal community structure shifts across forest age categories (10, 50, 100 years old) under conifers but not under broadleaf trees. In particular, *Tuber* abundance is consistently higher under broadleaf trees than conifers, while *Wilcoxina* presents the reverse trend [28]. *Tuber melanosporum* mycorrhization can increase leaf photosynthetic rate and promote the nutrient acquisition of broadleaf trees (e.g., *Quercus mongolica*) [33]. Pinaceae genera preferably host specific ECM fungal taxa [34]. For instance, *Picea* is strongly associated with *Amphinema*, *Hygrophorus*, and *Lactarius*; *Pinus* with *Wilcoxina*, *Rhizopogon*, and *Suillus*; and *Abies* with *Piloderma* and *Russula*.

In this study, we integrated neutral theory modeling, alpha/beta diversity analyses, and random forest (RF) algorithms to test the following two hypotheses: (1) EcM fungal community assembly driven by specific taxa would shift from niche-based to neutral processes as host trees mature, due to the development of mycorrhizal networks and reduced dispersal limitation; and (2) coniferous hosts with recalcitrant litter, would intensify niche filtering in juveniles and enhance network connectivity in adults, whereas broadleaf hosts would promote stochasticity via high-quality organic inputs. By resolving these dynamics, our work aimed to advance the mechanistic understanding of EcM fungal assembly as influenced by host age and identity.

## 2. Materials and Methods

### 2.1. Study Site and Experimental Design

This study was carried out in the National Nature Reserve on the northern slope of Changbai Mountain, located in northeastern China (41°43′ N to 42°26′ N, 127°42′ E to 128°17′ E). Established in 1960, the reserve covers 200,000 ha, with elevation ranging from 740 m to 2691 m. This region has a temperate continental monsoon climate. The annual average temperature is 2.8 °C, ranging from −13.7 °C (January) to 19.6 °C (July). The annual average precipitation is 700 mm, majorly occurring between July and September [1,35]. To represent variations in host age and identity, we selected juveniles and adults of three coniferous species including *Abies nephrolepis* (*Trautv.* ex Maxim.) *Maxim.* (smelly fir), *Picea jezoensis* (*Siebold & Zucc*.) Carrière (Yeddo spruce), and *Pinus koraiensis* (*Siebold & Zucc*) (*Korean pine*) and three broadleaf species including *Acer mono* (*Acer pictum subsp. mono* (Maxim.) *H. Ohashi*) (*Red maple*), *Betula platyphylla* (*Betula pendula subsp.* mandshurica (Regel) *Ashburner & McAll.* (*Betulaceae*)) (*Asian white birch*), and *Quercus mongolica* Fisch. ex Ledeb. (*Fagaceae*) (*Mongolian oak*). Fir and spruce were sampled at elevations of 1000–1100 m in a secondary coniferous-broadleaf mixed forest, and pine was studied at elevations of 800–900 m in a broadleaf *Korean pine* mixed forest. Maple was found at elevations of 800–900 m in a broadleaf *Korean pine* mixed forest, birch at elevations of 850–950 m in a secondary poplar–birch forest, and oak at elevations of 900–1000 m in a secondary coniferous–broadleaf mixed forest. Plant individuals were classified into two age groups according to diameter at breast height (DBH): juveniles (20–30 years, DBH 10–20 cm) and adults (>70 years, DBH > 30 cm), which represent the stem exclusion (intense competition for resources) and stand reinitiation (senescence initiation and canopy gap formation) stages [26,36,37], respectively.

### 2.2. Sampling and Molecular Analysis

In July 2019, we generated coordinates within the host species’ distribution range and used a stratified random sampling procedure to select 10 plant individuals (at least 50 m apart) from each growth stage at each site. Fine roots were collected by indexing taproots, excavated in three distinct directions, and subsequently combined into a composite sample. A total of 120 root samples (6 tree species × 2 stages × 10 replicates) were obtained. Samples were transported on ice and stored at −20 °C.

Before the molecular analysis, residual soil was washed off roots with running tap water. Approximately 400 root tips were randomly obtained from each sample under a stereomicroscope and ground into powder with the aid of liquid nitrogen. Genomic DNA was extracted from 0.5 g of root powder via the FH Plant DNA Kit (Beijing Demeter Biotech Co., Ltd., Beijing, China). DNA quality and concentration were measured via the optical density at 260/280 nm on a Thermo Scientific NanoDrop 2000 spectrophotometer (Thermo Scientific, Waltham, MA, USA), and values ranging from 1.8 to 2.0 were selected for downstream polymerase chain reaction (PCR) amplification.

### 2.3. PCR Amplification and Illumina Sequencing

The internal transcribed spacer 2 (ITS2) region was amplified from the extracted DNA via primers ITS3tagmix (5′-CATCGATGAAGAACGCAG-3′) and ITS4NGS (5′-TCCTSCGCTTATTGATATGC-3′) [38]. PCRs were performed in 25 µL reactions containing 2.5 μL of DNA template, 12.5 μL of KAPA HiFi HotStart ReadyMix (Thermo Scientific, Waltham, MA, USA), and 5 μL of each primer. The amplification program consisted of an initial denaturation at 95 °C for 3 min, followed by 25 cycles of 95 °C for 30 s, 55 °C for 30 s, and 72 °C for 30 s, with a final extension at 72 °C for 5 min. A negative control with nuclease-free water was included to ensure successful amplification.

Nextera XT Index N7 and S5 (Illumina, San Diego, CA, USA) were employed as the forward and reverse primers, respectively, for the second round of PCR amplification. The 50 μL reaction mixtures comprised 5 μL of DNA, 10 μL of PCR-grade water, 25 μL of KAPA HiFi HotStart ReadyMix, and 5 μL of each index primer (N7xx/S5xx). The reaction was initiated at 95 °C for 3 min, followed by 8 cycles of 95 °C for 30 s, 55 °C for 30 s, and 72 °C for 30 s, with a final extension at 72 °C for 5 min. PCR products were purified with AMPure magnetic beads (Beckman Coulter Life Sciences, Indianapolis, IN, USA) and pooled at equimolar concentrations for amplicon library preparation. Sequencing was conducted on an Illumina MiSeq platform (Illumina, San Diego, CA, USA) at the Testing and Analysis Center, Institute of Applied Ecology, Chinese Academy of Sciences.

### 2.4. Sequence Processing

Raw paired-end reads generated from Illumina sequencing were further processed via QIIME2 2020.11 [39]. The DADA2 method [40] was used for quality filtering, denoising, and chimera removal, generating amplicon sequence variants (ASVs). Paired-end sequences were merged after denoising, with low-quality ends (3′) trimmed at reverse reads. Taxonomic assignment was performed using q2-feature-classifier on the UNITE+INSDC ITS database (version 8.3) at 99% sequence similarity [41]. We excluded ASVs that merely occurred in a single sample to avoid artifactual ASVs, as well as those with less than 10 sequences in a given sample to minimize false presences [42]. EcM fungal taxa were identified based on genus-level functional annotations in the Fungaltraits database, with >90% similarity to a fungal Species Hypothesis (SH) with known ecological function [43]. Due to uneven sequence depths, samples were rarefied to a minimum sequence depth of 23,167 using the rarefy_even_depth function in R 4.2.0 (R Development Core Team 2022). This threshold was chosen to ensure sufficient statistical power for downstream analyses, as recommended in previous studies [44,45]. The raw sequencing data were deposited in the NCBI Sequence Read Archive (SRA) (BioProject ID: PRJNA1051329).

### 2.5. Statistical Analyses

All statistical analyses were performed using R 4.2.0 (R Development Core Team 2022). To evaluate the role of neutral processes in EcM fungal community assembly, we adapted a generalized additive model (GAM)-based framework to analyze the relationship between species occurrence frequency and log10-transformed mean relative abundance across host developmental stages (juveniles vs. adults) and species identities (conifers vs. broadleaves) by using the mgcv R package. Log10 transformation was applied to normalize the distribution of relative abundances.

Neutral processes were assessed by fitting a GAM to predict species occurrence frequency as a nonlinear function of mean relative abundance. The fit of the neutral model was quantified using a pseudo-R^2^ (1 − residual deviance/null deviance), where higher values indicate greater explanatory power of neutral processes. While pseudo-R^2^ is a useful measure of model fit, it is important to note that it does not directly assess the proportion of variance explained in the same way as traditional R^2^ in linear models. As such, caution should be exercised when interpreting the magnitude of pseudo-R^2^ values in the context of our analyses, especially given the complex, nonlinear nature of the GAM framework used in this study. To estimate dispersal dynamics, we calculated the migration rate (m) as the ratio of observed-to-predicted variance in species occurrence frequencies:

m = ∑(∫obs⋅(1 − ∫obs))/∑(∫ pred⋅(1 − ∫pred))(1)

where ∫obs is the observed occurrence frequency and ∫pred is the GAM-predicted occurrence frequency. In this framework, the following terms are defined as:

- High m values (m→1) indicate strong mismatch between observed and neutral expectations, reflecting increased migration rates or greater dynamic variability in species distribution.
- Low m values (m→0) suggest closer alignment with neutral predictions, implying a increased dispersal limitation.

This approach builds on variance-partitioning methods used in microbial ecology [46] but adapts them for EcM fungi by explicitly modeling nonlinear abundance–occupancy relationships.

Alpha diversity was assessed using Shannon, Simpson, Pielou’s evenness, and observed richness indices, calculated with diversity and specnumber functions from the vegan R package. A permutational multivariate analysis of variance (PERMANOVA) and an analysis of similarities (ANOSIM) were used to test for significant compositional differences between groups. Dissimilarity in fungal community structure was calculated based on the Bray–Curtis distance between tree ages or types, using Hellinger-transformed relative abundance data. The confidence intervals for each stage group and tree type were determined from non-metric multidimensional scaling (NMDS) ordination by the stat_ellipse function in the vegan R package [47]. RF algorithms were used to identify fungal taxa most strongly associated with specific host age and type with the random forest R package [48]. Differences in EcM fungal communities between groups were examined via a one-way analysis of variance (ANOVA) or the nonparametric Kruskal-Wallis test in cases of non-normality (Levene test) and the heterogeneity of variance (Bartlett test) [49].

## 3. Results

### 3.1. Neutral Processes Dominated More in Adults than Juveniles, Modulated by Host Identity

**Figure 1:**
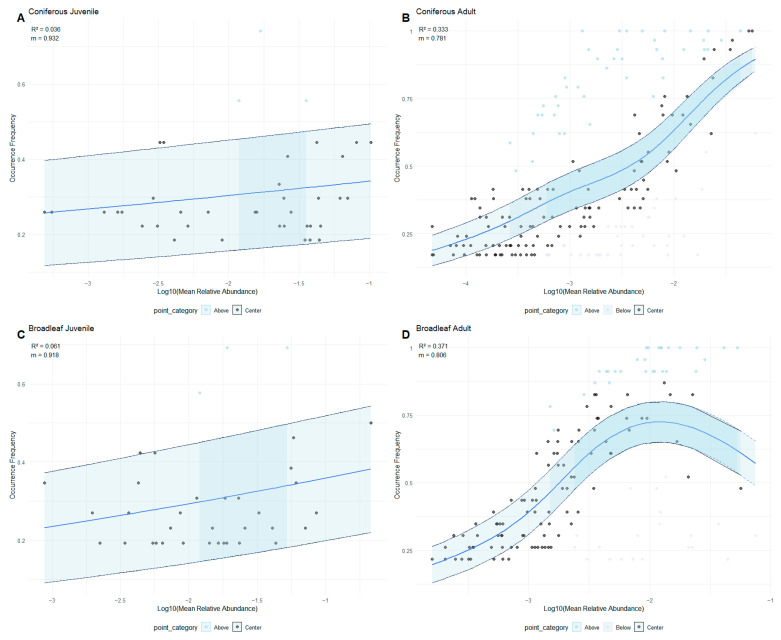
Neutral model fit for EcM fungal community assembly across host developmental stages (juvenile vs. adult) and identities (coniferous vs. broadleaf). (**A**) Coniferous juvenile; (**B**) Coniferous adult; (**C**) Broadleaf juvenile; (**D**) Broadleaf adult. Solid lines represent model predictions; shaded areas indicate 95% confidence intervals. R^2^ values (pseudo-R^2^) and migration rates (m) are shown for each group. The middle blue line represents the best fit of the neutral model, and the side blue line represents the 95% confidence interval around the model prediction.

Root EcM fungal communities presented distinct assembly patterns depending on both host developmental stage and species identity (Figure 1). Juveniles displayed the characteristics of niche-driven assembly, with minimal fit to the neutral model (R^2^ equaled 0.036 and 0.061 for conifers and broadleaves, respectively). Meanwhile, juveniles showed high m values (0.932 and 0.918 for conifers and broadleaves, respectively), indicating strong dispersal dynamics. At the adult stage, however, the R^2^ values were 0.333 for coniferous and 0.371 for broadleaf trees. While niche-based processes may still play a significant role, neutral processes explained 33–37% of community variation in adults, suggesting their increased influence during late succession. Well-established mycorrhizal networks of adult hosts facilitated greater stability and reduced dispersal rates in EcM fungal communities, with m values of 0.781 for coniferous and 0.806 for broadleaf sets. The assembly patterns of fungal communities were modestly influenced by host identity. Broadleaf adult sets exhibited stronger neutral signature and dispersal dynamic compared to coniferous adult sets. Coniferous juvenile systems exhibited the weakest fit to the neutral model and the highest dispersal dynamics, suggesting stronger environmental filtering and greater dynamics variability in species distribution than broadleaf juvenile systems.

### 3.2. Alpha Diversity Increased with Host Maturity, Moderated by Host Identity

**Figure 2:**
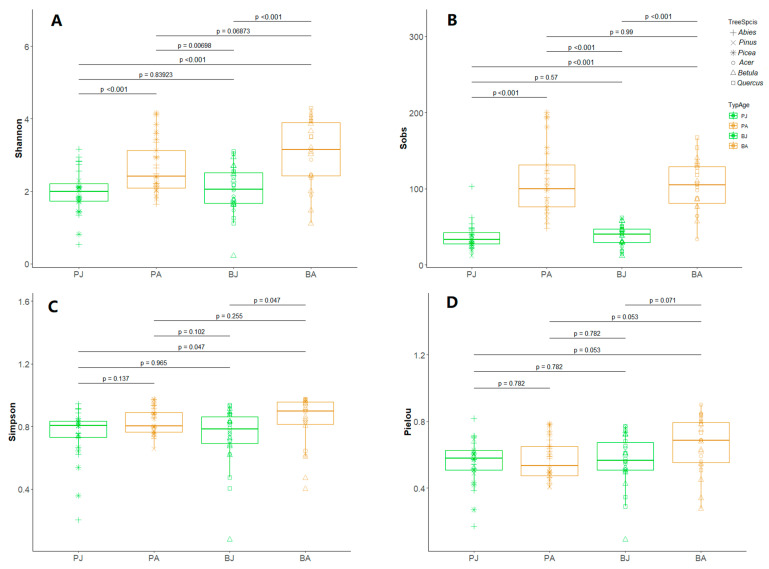
Alpha diversity of EcM fungal communities colored by host developmental stage (juvenile/adult) and shaped by host identity (coniferous/broadleaf). (**A**) Shannon diversity; (**B**) Observed richness (Sobs); (**C**) Simpson diversity; (**D**) Pielou’s evenness. Pinaceae juvenile, PJ; Pinaceae adult, PA; Broadleaf juvenile, BJ; Broadleaf adult, BA. Differences are examined via one-way analysis of variance (ANOVA) or the nonparametric Kruskal-Wallis test in cases of non-normality (Levene test) and heterogeneity of variance (Bartlett test).

Adult trees harbored significantly higher EcM fungal diversity than juvenile trees (Figure 2). Shannon diversity increased with host aging by 34% and 50% in coniferous and broadleaf stands, respectively (*p* < 0.001, Figure 2A). Adult broadleaf stands (3.067 ± 0.189; mean ± se) had marginally higher Shannon diversity than adult coniferous stands (2.658 ± 0.136) (*p* = 0.069), though host identity had no significant effect on Shannon diversity at the juvenile stage (conifers: 1.989 ± 0.117; broadleaves: 2.040 ± 0.128) (*p* > 0.05). This indicates niche diversification in well-established root systems. EcM fungal species richness (Sobs) also mirrored an age-driven accumulation trend, with adult trees supporting 1.7–1.9 times more taxa than juveniles (104–109 vs. 37–38 taxa) (*p* < 0.001, Figure 2B). Host identity did not significantly affect fungal richness at either stage (*p* > 0.05, Figure 2B). The Simpson diversity and Pielou’s evenness indices further supported these findings, with adult broadleaf hosts exhibiting higher evenness than juvenile stands (*p* < 0.1, Figure 2C,D), reflecting a more balanced community composition with reduced dominance by a few taxa.

### 3.3. Beta Diversity Varied with Host Maturity and Species Identity

**Figure 3:**
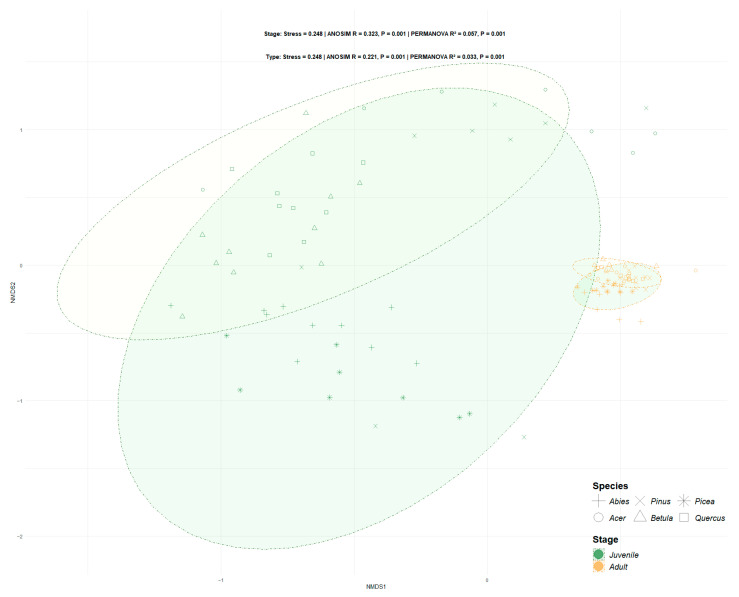
Non-metric multidimensional scaling analysis of EcM fungal communities, colored by host developmental stage (juvenile/adult) and shaped by host identity (coniferous/broadleaf). Ellipses represent 95% confidence intervals.

NMDS (stress = 0.248) revealed clear compositional shifts in EcM fungal communities, driven by both host developmental stage and species identity (Figure 3). PERMANOVA indicated that developmental stage explained 5.7% of the variance (*p* = 0.001), while host identity accounted for 3.3% (*p* = 0.001), highlighting stronger age-related effects than host identity effects. ANOSIM further corroborated this trend, showing moderate dissimilarity between stage groups (R = 0.323, *p* = 0.001) and weaker difference between host types (R = 0.221, *p* = 0.001).

### 3.4. Stage-Specific Fungal Taxa

**Figure 4:**
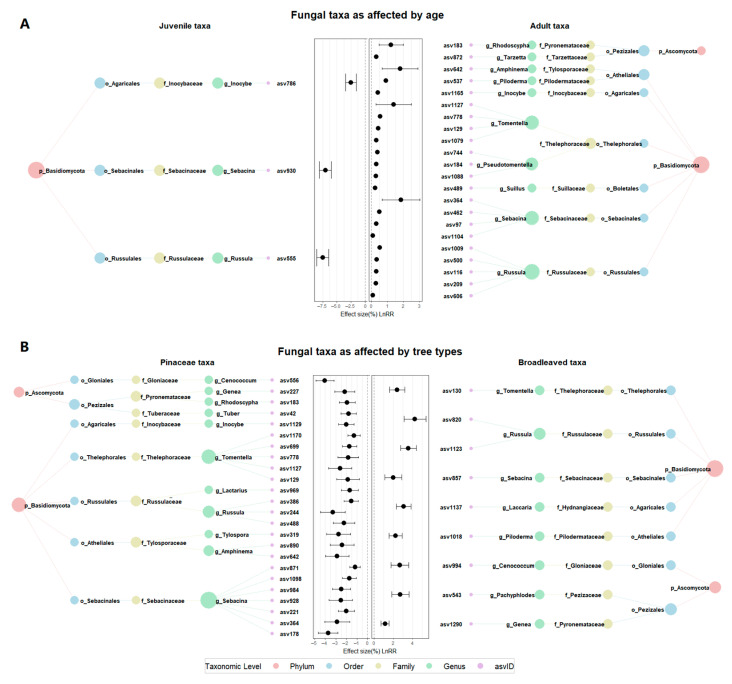
Random forest classification of EcM fungal taxonomy associated with (**A**) host developmental stage (juvenile vs. adult) and (**B**) host identity (coniferous vs. broadleaf). Models are trained on 1000 trees using the random Forest R package. Dots represent means, error bars exhibit 95% confidence intervals. Log-transformed response ratios are abbreviated as LnRR. Dashed lines represent the effect size (lnRR = 0). Effects of host developmental stage and identity are significant *p* < 0.05. Taxonomic levels are abbreviated as follows: Phylum (*p*), Order (o), Family (f), Genus (g), and amplicon sequence variants (ASV).

RF analysis identified ASVs that were significantly associated with juvenile or adult hosts (Figure 4A). ANOVA confirmed these associations, with several ASVs showing marked differences in relative abundance between stages (*p* < 0.01, Table 1). For instance, ASV786 (*Inocybe*, *Inocybaceae*; Genus, Family) was substantially more abundant in juvenile hosts (3.069 ± 1.538%) compared to adult hosts (0.1417 ± 0.0913%). ASV930 (*Sebacina*, Sebacinaceae) was found in juvenile hosts (0.1064 ± 0.0245%) but nearly absent in adult hosts (0.0007 ± 0.0007%)). Similarly, ASV555 (*Russula*, Russulaceae) was more abundant in juveniles (0.2329 ± 0.1441%) than in adults (0.0042 ± 0.0030%).

The taxa absent in juvenile but nearly exclusive to adult hosts included ASV209 (*Russula*, Russulaceae; 0.8983 ± 0.5476%), ASV1165 (*Inocybe*, Inocybaceae; 0.4652 ± 0.2546%), and ASV778 (*Tomentella*, Thelephoraceae; 0.2061 ± 0.1652%) (*p* < 0.001; Table 1). In addition, ASV183 (*Rhodoscypha*, Pyronemataceae), ASV642 (*Amphinema*, Tylosporaceae), ASV1127 (*Tomentella*, Thelephoraceae), and ASV364 (*Sebacina*, Sebacinaceae) were much more abundant in adult hosts than their juvenile counterparts (*p* < 0.01; Table 1).

### 3.5. Host-Specific Fungal Taxa

RF and ANOVA analyses also identified ASVs that were significantly associated with either coniferous or broadleaf hosts (Figure 4B; *p* < 0.05, Table 2). The taxa occurring in coniferous but rarely in broadleaf hosts were ASV556 (*Cenococcum*, Gloniaceae; 0.0543 ± 0.0181%), ASV1129 (*Inocybe*, Inocybaceae; 0.1515 ± 0.1048%), ASV699 (*Tomentella*, Thelephoraceae; 0.2445 ± 0.1347%), ASV386 (*Russula*, Russulaceae; 0.2761 ± 0.1801%), and ASV178 (*Sebacina*, Sebacinaceae; 0.6349 ± 0.4949%). In addition, the taxa with the relative abundances over 1% in coniferous hosts included ASV244 (*Russula*, Russulaceae), ASV319 (*Tylospora*, Tylosporaceae), ASV642 (*Amphinema*, Tylosporaceae), and ASV364 (*Sebacina*, Sebacinaceae). Conversely, broadleaf hosts harbored taxa such as ASV130 (*Tomentella*, Thelephoraceae) (1.758 ± 1.135%) and ASV820 (*Russula*, Russulaceae) (0.9874 ± 0.3098%) with high relative abundances, as well as ASV1123 (*Russula*, Russulaceae) (0.3256 ± 0.1549%) that was notably rare in coniferous trees.

## 4. Discussion

Our study reveals distinct shifts in EcM fungal community assembly and diversity, driven by host tree’s development. The low R^2^ values (<0.1) at juvenile stage suggest that fungal community assembly is primarily governed by deterministic processes, such as environmental filtering and niche-based recruitment. These findings align with patterns observed in forest succession, where juvenile trees—subject to stem exclusion and intense competition for resources (e.g., light)—favor niche-based fungal assembly [1,7,50,51]. Early-stage plant symbionts usually possess unique traits, such as great dispersal capacity, strong competitive ability, and highly specialized plant–fungus interactions, which permit their preferential recruitment and colonization in seedlings [6,7,35,52,53,54]. The absence of juvenile-specific taxa, combined with low alpha-diversity, suggests that only widespread taxa are able to survive in fiercely competitive and disturbance-prone niches. Great dispersal rates (m > 0.9) at the juvenile stage are probably because of limited and heterogeneous C input from rhizodeposits and leaf litter, as well as fragmented and undeveloped root systems of young trees [55]. In contrast, rhizospheric fungal assembly at the early stage of rice growth is chiefly driven by stochastic forces (dispersal limitation) [53]. Such divergent contribution between deterministic vs. stochastic processes might be ascribed to distinct soil nutrient status and vegetation composition between forests and rice fields. Specifically, the rice fields, characterized by more sufficient nutrients, may facilitate stochastic process-driven community assembly. Additionally, in monoculture systems, the uniformity of plant species may limit the deterministic processes associated with host-specific selection.

In contrast, adult trees displayed a stronger influence of neutral processes, with higher R^2^ values (>0.3) and lower m-values (<0.9). This transition from deterministic to stochastic community assembly reflects increased dispersal limitation and enhanced stability in fungal communities along with the development of well-established mycorrhizal networks. With host growth, the successional recruitment of new taxa, the formation of extensive mycorrhizal networks, and long-term biotic–abiotic interactions all contribute to high alpha-diversity and the decreasing role of ecological drift and dispersal in shaping EcM fungal communities [6,54]. It is believed that host maturity can stabilize the surrounding niche space (e.g., soil), supporting a higher richness of microbial taxa with distinct traits [6,35]. Stable niches under adult trees promote taxonomic specification, as evidenced by the presence of adult-specific taxa such as *Tomentella* and *Amphinema.* These taxa are linked to well-developed symbiotic networks and may facilitate stochastic processes by enhancing fungal connectivity [56,57,58]. Together, these findings partially support Hypothesis 1 but suggest that the transition from niche-based to neutral processes is not a clear-cut shift; rather, it is a gradual transition driven by the development of mycorrhizal networks and reduced dispersal dynamics. This highlights the dynamic interplay between niche-based and neutral processes throughout tree development. Specifically, juvenile trees are dominated by deterministic (niche-based) processes, with low fungal diversity and high specialization. In contrast, adult trees experience a shift towards neutral processes, marked by increased fungal diversity, the establishment of more complex mycorrhizal networks.

The tree species identity modulated the strength of neutral processes in adult trees, as evidenced by the variation in R^2^ values and dispersal dynamics observed between coniferous and broadleaf trees. The contrasting traits of conifer and broadleaf species present distinct selection pressures on fungal community composition [52,59,60]. Broadleaf adult sets exhibited the strongest stochastic dynamics (R^2^ = 0.371), probably due to their heterogeneous microhabitats and high-quality, diverse organic inputs, which buffer against deterministic selection pressures [61,62]. Their moderate dispersal limitation (m = 0.806) suggests balanced dispersal dynamics, probably driven by the typically more varied and potentially less cohesive mycorrhizal networks associated with broadleaf specie [63]. In contrast, adult pine trees showed slightly weaker neutral signature (R^2^ = 0.333) and the lowest dispersal dynamics (m = 0.781) compared to broadleaf trees. These patterns may arise from pine-specific traits, such as dense root systems and recalcitrant litter, which accelerate network formation and predominance of specific taxa, enhancing decomposition of recalcitrant organic materials [64,65]. Accordingly, the pine-specific taxa such as *Amphinema* (ASV642) and *Sebacina* (ASV364), which are also adult-specific, are known to be highly adapted to the rapid decomposition of recalcitrant litter [8]. These findings reflect niche partitioning and high specification in coniferous mycorrhizal networks.

At juvenile stage, broadleaf trees exhibited slightly higher neutral dynamics and dispersal limitation compared to conifers, suggesting weaker environmental filtering. This may be due to broader host compatibility for early-colonizing fungi in broadleaf systems, where more variable conditions allow a wider range of fungal taxa to survive [66,67]. In contrast, pine-specific exudates (e.g., diterpenes) may constrain the establishment of generalists (e.g., Sebacina, *Russula*) that are typically fast colonizers. This, in turn, increases niche partitioning within coniferous systems and promotes taxonomic specification [68]. Accordingly, conifer specialists such as *Cenococcum* and *Tylospora* likely exploit pine-specific exudates to gain competitive advantages [69,70]. The phylogenetic partitioning of EcM fungal taxa between coniferous and broadleaf trees not only reinforces the importance of host-derived niche filtering but also emphasizes the ecological roles of fungal specialization in forest ecosystems. The results strongly support Hypothesis 2, indicating that broadleaf trees promote stochasticity and weaker niche filtering due to their high-quality organic inputs. Conversely, coniferous trees intensify niche filtering in juveniles through their specialized exudates and foster network connectivity in adults, owing to traits such as recalcitrant litter and dense root systems.

This study uniquely explores how host developmental stage and vegetation type jointly influence EcM fungal community assembly, a relationship often overlooked in prior research. Our findings challenge the traditional binary view of niche-based vs. neutral processes, revealing instead a gradual transition mediated by mycorrhizal network development and host-driven niche stabilization. In addition, the observed niche filtering in juvenile trees suggests that early-stage restoration could benefit from inoculating stress-tolerant fungal taxa (e.g., Inocybe, *Sebacina*, and *Russula*) to support seedling establishment in nutrient-poor soils. In contrast, the stochastic dynamics under adult trees highlight the importance of preserving mature “nurse trees” or their mycorrhizal networks, which aid community stabilization of adult-specific taxa (*Tomentella*, *Rhodoscypha*, and *Amphinema*) in reforestation efforts. Tree species selection can be optimized based on succession goals: broadleaf species may promote stochastic assembly and organic matter turnover, while conifers are better suited for sites requiring niche specialization. By aligning forest management strategies with these host stage- and identity-dependent assembly rules, we can enhance the resilience of restored forests, promoting both short-term survival and long-term ecosystem complexity.

## 5. Conclusions and Implications

This study highlights the role of host age and identity as key drivers of EcM fungal community assembly. In mature hosts, neutral processes become more prominent due to well-established and stabilized mycorrhizal networks that limit stochastic dispersal. In contrast, juvenile trees are shaped by niche-based assembly, probably with host-specific root exudates, environmental filtering, and dispersal dynamics playing central roles. Host identity also modulates fungal community composition at both phylogenetic and functional levels, with coniferous and broadleaf trees selecting distinct functional guilds suited to their biogeochemical environments. These findings improve our understanding of fungal community assembly, emphasizing the dynamic balance between deterministic and stochastic processes across forest succession. Incorporating belowground biodiversity into forest management can enhance ecosystem resilience, boost fungal diversity, and support sustainable forest ecosystems amid global environmental change.

## Figures and Tables

**Table 1:** Effect of host developmental stage on the relative abundances of EcM fungal species.

ASVs	Species	Juvenile (%)	Adult (%)	*p* Values
ASV786	*Inocybe* sp.	3.069 ± 1.538 a	0.1417 ± 0.0913 b	<0.01
ASV555	*Russula* sp.	0.2329 ± 0.1441 a	0.0042 ± 0.0030 b	<0.001
ASV930	*Sebacina* sp.	0.1064 ± 0.0245 a	0.0007 ± 0.0007 b	<0.001
ASV116	*Russula vinosobrunneola*	0.6107 ± 0.3074 b	1.6185 ± 0.8141 a	<0.001
ASV364	*Sebacina* sp.	0.5149 ± 0.3411 b	1.3724 ± 0.5523 a	<0.001
ASV1009	*Russula* sp.	0.8488 ± 0.3788 b	0.9635 ± 0.5537 a	<0.001
ASV209	*Russula* sp.	0 ± 0 b	0.8983 ± 0.5476 a	<0.001
ASV489	*Suillus flavidus*	0.0068 ± 0.0049 b	0.7634 ± 0.3583 a	<0.001
ASV642	*Amphinema diadema*	0.5812 ± 0.3338 b	0.6917 ± 0.4717 a	<0.001
ASV1165	*Inocybe* sp.	0 ± 0 b	0.4652 ± 0.2546 a	<0.001
ASV1104	*Sebacina* sp.	0.2043 ± 0.1479 b	0.4614 ± 0.3157 a	<0.001
ASV129	*Tomentella* sp.	0.0012 ± 0.0012 b	0.4513 ± 0.3101 a	<0.001
ASV1127	*Tomentella* sp.	0.2043 ± 0.1865 b	0.4052 ± 0.1823 a	<0.001
ASV1088	*Pseudotomentella* sp.	0.0209 ± 0.0145 b	0.3359 ± 0.1813 a	<0.001
ASV778	*Tomentella* sp.	0 ± 0 b	0.2061 ± 0.1652 a	<0.001
ASV97	*Sebacina* sp.	0.0525 ± 0.0524 b	0.1479 ± 0.1343 a	<0.01
ASV744	*Pseudotomentella* sp.	0.0744 ± 0.0712 b	0.1437 ± 0.1039 a	<0.001
ASV183	*Rhodoscypha* sp.	0.0105 ± 0.0105 b	0.1412 ± 0.1020 a	<0.01
ASV537	*Piloderma* sp.	0.0912 ± 0.0393 b	0.1377 ± 0.1206 a	<0.05
ASV500	*Russula* sp.	0.0033 ± 0.0026 b	0.1207 ± 0.0148 a	<0.001
ASV872	*Tarzetta* sp.	0.0239 ± 0.0199 b	0.0824 ± 0.0496 a	<0.01
ASV184	*Pseudotomentella* sp.	0.0000 ± 0.0000 b	0.0759 ± 0.0108 a	<0.001
ASV606	*Russula* sp.	0.0007 ± 0.0007 b	0.0640 ± 0.0129 a	<0.001
ASV462	*Sebacina* sp.	0.0279 ± 0.0185 b	0.0572 ± 0.0515 a	<0.05
ASV1079	*Tomentella* sp.	0.0097 ± 0.0064 b	0.0543 ± 0.0481 a	<0.01

Differences are examined via one-way analysis of variance (ANOVA) or the nonparametric Kruskal-Wallis test in cases of non-normality (Levene test) and heterogeneity of variance (Bartlett test). Values are indicated as “% = mean relative abundance ± SE”. The different characters in a single row indicate significant differences between juvenile and adult stages. Amplicon sequence variant is abbreviated as ASV.

**Table 2:** Effect of host identity on the relative abundances of EcM fungal species.

ASV	Species	Pinaceae (%)	Broadleaved (%)	*p* Values
ASV364	*Sebacina* sp.	1.706 ± 0.5915 a	0.0637 ± 0.0193 b	<0.01
ASV642	*Amphinema diadema*	1.186 ± 0.5288 a	0.0070 ± 0.0020 b	<0.01
ASV319	*Tylospora* sp.	1.153 ± 0.5281 a	0.0391 ± 0.0276 b	<0.05
ASV244	*Russula* sp.	1.004 ± 0.4452 a	0.0400 ± 0.0196 b	<0.01
ASV488	*Russula* sp.	0.6762 ± 0.5568 a	0.0057 ± 0.0016 b	<0.05
ASV178	*Sebacina* sp.	0.6349 ± 0.4949 a	0.0006 ± 0.0004 b	<0.001
ASV890	*Amphinema* sp.	0.5575 ± 0.2705 a	0.0532 ± 0.0344 b	<0.05
ASV1127	*Tomentella* sp.	0.5529 ± 0.2401 a	0.0191 ± 0.0044 b	<0.05
ASV221	*Sebacina* sp.	0.4730 ± 0.2948 a	0.0018 ± 0.0017 b	<0.01
ASV928	*Sebacina* sp.	0.4667 ± 0.1670 a	0.0121 ± 0.0032 b	<0.01
ASV129	*Tomentella* sp.	0.4042 ± 0.2885 a	0.0183 ± 0.0062 b	<0.05
ASV984	*Sebacina* sp.	0.3016 ± 0.1803 a	0.0633 ± 0.0631 b	<0.01
ASV386	*Russula* sp.	0.2761 ± 0.1801 a	0.0003 ± 0.0002 b	<0.05
ASV699	*Tomentella* sp.	0.2445 ± 0.1347 a	0.0001 ± 0.0001 b	<0.05
ASV42	*Tuber californicum*	0.2357 ± 0.2231 a	0.0018 ± 0.0013 b	<0.05
ASV778	*Tomentella* sp.	0.1840 ± 0.1536 a	0.0085 ± 0.0029 b	<0.05
ASV1129	*Inocybe* sp.	0.1515 ± 0.1048 a	0.0003 ± 0.0002 b	<0.01
ASV183	*Rhodoscypha* sp.	0.1401 ± 0.0951 a	0.0011 ± 0.0007 b	<0.05
ASV227	*Genea* sp.	0.1344 ± 0.0431 a	0.0095 ± 0.0054 b	<0.05
ASV969	*Lactarius* sp.	0.1146 ± 0.0672 a	0.0023 ± 0.0012 b	<0.05
ASV556	*Cenococcum* sp.	0.0543 ± 0.0181 a	0.0000 ± 0.0000 b	<0.001
ASV1098	*Sebacina* sp.	0.0454 ± 0.0198 a	0.0003 ± 0.0003 b	<0.05
ASV1170	*Tomentella* sp.	0.0164 ± 0.0131 a	0.0004 ± 0.0003 b	<0.05
ASV871	*Sebacina* sp.	0.0074 ± 0.0032 a	0.0000 ± 0.0000 b	<0.05
ASV130	*Tomentella* sp.	0.0049 ± 0.0040 b	1.758 ± 1.135 a	<0.01
ASV820	*Russula* sp.	0.0336 ± 0.0175 b	0.9874 ± 0.3098 a	<0.001
ASV1123	*Russula* sp.	0.0000 ± 0.0000 b	0.3256 ± 0.1549 a	<0.001
ASV857	*Sebacina* sp.	0.0641 ± 0.0375 b	0.1185 ± 0.0756 a	<0.05
ASV543	*Pachyphlodes nemoralis*	0.0121 ± 0.0108 b	0.1033 ± 0.0481 a	<0.01
ASV994	*Cenococcum* sp.	0.0076 ± 0.0033 b	0.0902 ± 0.0415 a	<0.01
ASV1137	*Laccaria parva*	0.0012 ± 0.0012 b	0.0701 ± 0.0306 a	<0.001
ASV1018	*Piloderma* sp.	0.0002 ± 0.0002 b	0.0463 ± 0.0188 a	<0.01
ASV1290	*Genea zamorana*	0.0000 ± 0.0000 b	0.0018 ± 0.0009 a	<0.01

Differences are examined via one-way analysis of variance (ANOVA) or the nonparametric Kruskal-Wallis test in cases of non-normality (Levene test) and heterogeneity of variance (Bartlett test). Values are indicated as “% = mean relative abundance ± SE”. The different characters in a single row indicate significant differences between Pinaceae and Broadleaf species. Amplicon sequence variant is abbreviated as ASV.

## Data Availability

The dataset and associated R codes used in the main results are available upon reasonable request to the corresponding author.
